# Book Review: Quiet: The Power of Introverts in a World That Can't Stop Talking

**DOI:** 10.3389/fpsyg.2017.00155

**Published:** 2017-02-07

**Authors:** Lesley A. Clack

**Affiliations:** Department of Health Sciences, Armstrong State UniversitySavannah, GA, USA

**Keywords:** introversion, extraversion, personality, leadership, communication

“In a gentle way, you can shake the world.”—Mahatma Gandhi

This quote is an excellent reflection of the author's focus in the book “*Quiet: The Power of Introverts in a World that Can't Stop Talking*,” by Susan Cain. The author discusses her view that introverts are highly undervalued, particularly in leadership positions. She holds the belief that extroverts are rated as “smarter, better-looking, more interesting, and more desirable,” and that introversion is considered a “second-class personality trait” (Cain, 2013). Her perspective originates from her own self-proclaimed status as an introvert and her experiences in the workplace. A common perception does exist that extroverts are the most effective communicators, and thus, make the best leaders (Bradley and Hebert, 1997). Research studies throughout time have consistently reaffirmed the belief that extroverts are more likely to emerge as leaders, and are more likely to be perceived as effective (Grant et al., 2011). Cain looks to dispel that belief and make an argument for the importance of introverts as leaders. This book is a great read for introverted individuals aspiring to become leaders, and for organizations seeking knowledge on how to provide a conducive environment in which introverted leaders can be successful. This work has important implications for many fields that are heavily dependent upon good leaders, as great emphasis has traditionally been placed on the importance of extraverted characteristics for leadership success.

The book was divided into four parts. Part One, “The Extrovert Ideal,” focuses on this concept that the author defines as “the omni-present belief that the ideal self is gregarious, alpha, and comfortable in the spotlight.” She discusses the historical creation of the “Culture of Personality” that shapes our view of others. The author gives examples of how organizations, such as Harvard Business School, seemingly try to turn introverts into extroverts by equating such characteristics as speaking up in class with performance. The author also discusses positive characteristics that are attributed to introverts, such as creativity, and whether that is a true reflection. This section leads one to ponder the ethics of placing such attributions on individuals, whether introverted or extraverted, and the influence that can have on an individual's success.

Part Two is entitled “Your Biology, Your Self?” In this section, the author discusses the connection between temperament and personality, and studies that have examined the influences of innate, inborn temperament on personality type. She uses scientific evidence to explain her so-called “rubber band theory” of personality, meaning that we are elastic and can stretch ourselves beyond our innate traits, but only within certain limits. The author also discusses the trade-off theory and the things that are lost or gained by being either an introvert or extrovert. Warren Buffett is used as an example of an individual that used his introvert qualities to his benefit to become successful and powerful.

Part 3, “Do All Cultures Have an Extrovert Ideal?” examines the idea that the Extrovert Ideal is an American standard that is not typical in other cultures. The author states that other cultures do not emphasize traits, such as class participation, as a measure of success. Gandhi is discussed as an example of a classic introvert that found power in his shyness. This leads one to think about whether we, as a society, focus on personality traits too much as a measure of an individual's capability of success in certain work environments.

Part 4, “How to Love, How to Work,” discusses the idea that we shift our personality traits based on the situation that we find ourselves in. The author also discusses the attraction between individuals of opposite personality types, as well as the potential difficulties in communication between introverts and extroverts. The author concludes with a discussion on how to foster traits such as depth and sensitivity, rather than trying to force introverted children to be extraverted.

In the Conclusion, entitled “Wonderland,” the author urges readers to be true to their self, and to put themselves in situations that play well with their personality, rather than forcing uncomfortable situations. As she eloquently puts, managers should “make the most of introverts' strengths—these are the people who can help you think deeply, strategize, solve complex problems, and spot canaries in your coal mine.” These are definitely qualities that are valued in leaders in many fields. This book offers new insights and will serve as a valuable source of information for management professionals. In addition, it helps to shatter the belief that extroverted individuals are superior and provides a much needed change in perception for introverts hoping to become leaders.

## Author contributions

The author confirms being the sole contributor of this work and approved it for publication.

### Conflict of interest statement

The author declares that the research was conducted in the absence of any commercial or financial relationships that could be construed as a potential conflict of interest.
